# Amorphous Nanosuspensions Aggregated from Paclitaxel–Hemoglobulin Complexes with Enhanced Cytotoxicity

**DOI:** 10.3390/pharmaceutics10030092

**Published:** 2018-07-13

**Authors:** Chao Qin, Xiaofei Xin, Xue Pei, Lifang Yin, Wei He

**Affiliations:** Department of Pharmaceutics, School of Pharmacy, China Pharmaceutical University, Nanjing 210009, China; 1020142415@cpu.edu.cn (C.Q.); 15311010037@stu.cpu.edu.cn (X.X.); xuepeicpu@gmail.com (X.P.)

**Keywords:** nanosuspensions, amorphous, affinity, drug-protein complexes, cytotoxicity

## Abstract

Amorphous nanosuspensions (ANSs) enable rapid release and improved delivery of a poorly water-soluble drug; however, their preparation is challenging. Here, using hemoglobin (Hb) as a carrier, ANSs aggregated from paclitaxel (PTX)–Hb complexes were prepared to improve delivery of the hydrophobic anti-cancer agent. An affinity study demonstrated strong interaction between Hb and PTX. Importantly, the complexes could aggregate into <300 nm ANSs with high drug loading, which acidic condition facilitated their formation. Furthermore, the ANSs possessed improved cytotoxicity against cancer cells over the crystalline nanosuspensions. Taken together, ANSs aggregated from PTX–Hb complexes were developed, which could kill cancer cells with high efficiency.

## 1. Introduction

Over 40% of the marked products and approximately 90% of new drug candidates are poorly water-soluble compounds based on the biopharmaceutical classification system (BCS) [1]. Nanotechnology is one of the most commonly exploited approaches to enhancing the solubility and bioavailability of insoluble drugs [2,3]. Nanosuspensions of insoluble drugs, in which the drug can exist in crystalline or amorphous form, are a promising nanomedicine, possessing merits including extremely high drug-loading capacity, easy preparation, enhanced dissolution rate and saturation solubility, reproducibility, improved dose-bioavailability proportionality and increased patient compliance [4,5,6]. In contrast with crystalline nanosuspensions, amorphous nanosuspensions (ANSs) with unordered arrangement of molecules allow for faster dissolution rate and higher solubility [7,8,9].

Recently, it was reported that the nanocrystals were well uptaken by KB and HT-29 cells; however, it takes several hours to dissolve the intracellular crystals [10,11]. As is well known, the slowed drug release in cells could discount the treatment effect on cancer because keeping low drug concentration over time would induce multidrug resistance. In previous reports [12,13], using denatured soy protein isolate as a stabilizer, a nanosuspension formulation combining crystalline and amorphous drug was developed using a technique whereby the drug-protein complexes coated the nanocrystals of the insoluble drug. Inspired by these findings, we hypothesized that ANSs could be prepared via the aggregation of drug-protein complexes and were able to kill cancer cells with higher efficiency for rapid intracellular release post internalization.

Hemoglobin (Hb)—having four globular polypeptide subunits and a molecular weight of 64.5 kDa—is an abundant blood protein [14]. Hb can transport oxygen to the lung efficiently, as well as other organs, and is therefore regarded as an ideal oxygen carrier. On the other hand, paclitaxel (PTX) is one of the more frequently used anti-cancer drugs, acting through suppressing the microtubule dynamic instability and the resultant cell division [15]. Due to its extremely low water-solubility, a surfactant, Cremophor EL, has to be formulated into the marked product, Taxol, to enhance its solubility. However, the addition of Cremophor EL could induce allergenicity and toxicity to the body and thus limit PTX’s clinical use [16]. Consequently, Cremophor EL-free formulation for PTX is desired. With the ability to bind with hydrophobic agent and high biocompatibility, herein, Hb was used as the carrier to prepare drug–Hb complexes and ANSs with improved delivery of PTX.

## 2. Materials and Methods

### 2.1. Materials

Human hemoglobin (H7379, Hb), β-lactoglobulin (L3908, β-LG), fluorescein isothiocyanate isomer I (F7250, FITC), 3-(4,5-dimethylthiazol-2-yl)-2,5-diphenyltetrazolium bromide (MTT) and sodium azide (NaN_3_) were purchased from Sigma-Aldrich (St. Louis, MO, USA). Paclitaxel (PTX) was from Yew Biotechnology Co., Ltd. (Suzhou, China). Taxol was obtained from Bristol-Myers Squibb Investment Co., Ltd. (Shanghai, China). Lyso-tracker red, Annexin V-FITC/PI staining kit and 4′,6-diamidino-2-phenylindole (DAPI) were purchased from Beyotime Biotechnology (Shanghai, China). Cytochalasin D (Cy-D), nystatin, chlorpromazine (CPZ), methyl-β-cyclodextrin (M-CD), nocodazole (NOD), monensin (MON) and deoxyglucose (DG) were obtained from Aladdin Biochemical Technology Co., Ltd. (Shanghai, China). Fetal bovine serum (FBS), RPMI 1640, and phosphate buffer saline (PBS) were purchased from Thermo Fisher Scientific, Inc. (Waltham, MA, USA).

### 2.2. Preparation

PTX–Hb complexes were prepared by an anti-solvent precipitation method described in a previous report [17]. Briefly, a certain amount of PTX and 10 mg of Hb were dissolved in 1 mL of acetone and 10 mL of distilled water, respectively. Then PTX acetone solution was slowly added to the Hb solution with vigorous stirring in ice bath, followed by treatment in an ultrasonic probe (20–25 kHz, Scientz Biotechnology Co., Ltd., Ningbo, China) at 350 W for 15 min and evaporation under reduced pressure conditions.

FITC-labelled complexes of ANSs (FITC-ANSs) were prepared using a similar process, except that FITC was conjugated with the protein prior to mixing with the organic phase.

Crystalline nanosuspensions of PTX, β-LG-coated PTX nanocrystals (LPNs), and FITC-labelled LPNs (FITC-LPNs) were prepared by an anti-solvent precipitation procedure described in a previous report [18].

### 2.3. Characterization

Hydrodynamic diameter, zeta potential and polydispersity index were determined using a 90Plus Particle Size Analyzer (Brookhaven Instruments, Holtsville, NY, USA). Before determination, the sample was diluted approximately 10-fold.

Scanning electron microscopy (SEM, Hitachi, Tokyo, Japan) was used to investigate the morphology of ANSs. After centrifugation at 100,000× *g* for 10 min at 4 °C (Beckman Coulter Optima L-80XP, Brea, CA, USA), the precipitation was collected and diluted 100-fold. Then one drop of the sample was placed on the surface of a silicon chip, left overnight to evaporate the water at 25 °C, followed by coating with gold under vacuum condition and SEM examination at an excitation voltage of 10 kV.

### 2.4. Fluorescence Spectra

The fluorescence spectra were recorded with a fluorescence spectrometer (SHIMADZU RF-5301PC, Kyoto, Japan) with an emission spectrum of 300−500 nm and excitation wavelength of 295 nm. The resolutions of the emission and excitation were 5 nm and 15 nm, respectively. The protein concentration in pH 7 PBS was 1 mg/mL.

### 2.5. Circular Dichroism (CD) Spectra

CD spectra were recorded in a J-810 spectrometer (Tokyo, Japan) with a temperature-controlling unit and a quartz cuvette. The ellipticity was expressed in millidegrees. The analysis conditions were as follows: Bandwidth, 1 nm; response, 1 s; wavelength range, 250−190 nm; scan rate, 100 nm/min; cell length, 0.1 cm; temperature, 25 °C; protein concentration, 1 mg/mL (pH 7.0).

### 2.6. Affinity Study

The affinity of drug-protein was determined via the parameters including fluorescence quenching rate constant (*K*_q_), number of binding site (*n*), and binding constant (*K*_a_).

### 2.7. Powder X-Ray Diffraction (PXRD) and Differential Scanning Calorimetry (DSC)

PXRD was performed in an X’Pert PRO diffractometer (Panalytical, Holland) under the following conditions: 2θ range, 3–50°; san rate, 1°/min; step size, 0.02°; step time, 1 s. The patterns were collected at 40 kV and 60 mA with Cu Kα radiation (λ = 0.154 nm).

DSC analysis was conducted on a TA-Q2000 differential scanning calorimeter (TA Instruments, New Castle, DE, USA). A fixed weight (5 mg) of sample was placed in an aluminum pan and sealed. The samples were heated from 30 to 300 °C at a heating rate of 10 K/min. The instrument was calibrated with indium. All DSC analyses were performed in nitrogen atmosphere at a flow rate of 100 mL/min.

### 2.8. In Vitro Release

The drug release in vitro was investigated using a dialysis method. ANSs with 5 mg PTX and Taxol were transferred into a dialysis bag (molecular weight cut-off, 3500 Da) and dialyzed with continuous shaking at 120 rpm in pH 7.4 PBS containing 1% Tween 80 (*w*/*v*) at 37 °C. At specific time points, 1 mL of the dialysis solution was sampled and replaced with 1 mL fresh medium. The samples were then qualified by a high performance liquid chromatography (HPLC) system (SHIMAZU LC-10AT, Tokyo, Japan) according to a previous report [17].

### 2.9. Flow Cytometry (FCM)

For quantification of cellular uptake, FITC-labelled nanoparticles were incubated with A549 cells at 2.5 μg/mL of FITC at 37 °C or 4 °C. A fixed time later, the cells were washed three times, trypsinized and determined by FCM (BD FACSCalibur, Franklin Lakes, NJ, USA).

To investigate the endocytosis mechanism, A549 cells were pretreated with different uptake inhibitors at 37 °C for 30 min before incubation with ANSs. The inhibitors included Cy-D (10 μg/mL), nystatin (10 μM), CPZ (10 μg/mL), M-CD (2.5 nM), NOD (20 μM), MON (200 nM), NaN_3_ (10 nM) + DG (50 nM).

An apoptosis test in A549 cells was performed using Annexin V-FITC/PI staining kits and FCM. The cells were treated with different formulations with a fixed PTX concentration of 10 μg/mL for 48 h at 37 °C. Then the cells were trypsinized, harvested, stained with the kits according to the manufacturer’s protocol, and analyzed by FCM.

### 2.10. Confocal Imaging

A549 cells (1 × 10^5^) were seeded in confocal dishes and allowed to adhere for 24 h. FITC-labelled nanoparticles were incubated with A549 cells at 2.5 μg/mL of FITC at 37 °C for 4 h. Then the cells were washed three times with PBS and incubated with lyso-tracker-red for 2 h at 37 °C. Finally, the cells were rinsed three times again with PBS and observed using a confocal laser scanning microscope (CLSM, LSM700, Carl Zeiss, Oberkochen, Germany).

### 2.11. Cytotoxicity

4T1 and A549 cell viabilities against nanoparticles were determined by MTT assay. Cells (1 × 10^5^) and nanoparticles were added to each well, followed by incubation for 48 h at 37 °C. Then the treated cells were incubated with RPMI 1640 media containing 20 μL of MTT (5 mg/mL) for 4 h. Following removal of the media, DMSO was added to dissolve the violet crystals for absorbance measurement at 570 nm using a microplate reader (Multiskan, FC, Woburn, MA, USA).

### 2.12. Statistical Analysis

One-way analysis of variance was performed to assess the statistical significance of the differences between samples. The results are expressed as the means ± standard deviation. Significant differences were set as *p* < 0.05.

## 3. Results and Discussion

### 3.1. Drug-Protein Complex

**Figure 1:**
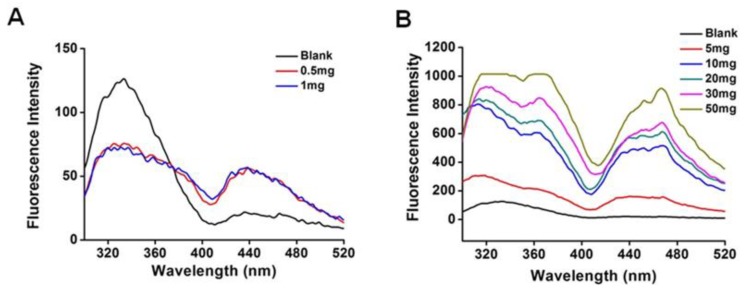
The fluorescence emission spectrum of amorphous nanosuspensions (ANSs) with paclitaxel (PTX) from 0–1 mg (**A**) and from 5–50 mg (**B**).

Local information regarding conformational changes in a protein resulting from its interaction with other agents can be detected using intrinsic fluorescence spectra. The maximal fluorescence at the excitation wavelength of approximately 340 nm is predominantly generated by the Trp residues surrounded by the hydrophobic residues of proteins. The fluorescence emission spectra of ANSs with different drug loading are shown in Figure 1. At PTX loading less than 1 mg, the maximum fluorescence intensity at 340 nm decreased significantly for drug incorporation (Figure 1A); by contrast, the maximum fluorescence increased with an increase in drug loading from 5 to 50 mg (Figure 1B). The fluorescence quenching at low drug loading suggested the formation of a protein-drug complex [19,20]. The increased fluorescence at high drug loading indicated the Trp residues were surrounded in a more hydrophobic environment and, consequently, demonstrated the aggregation of protein-drug complex [12].

To further quantify the affinity between the drug and protein, the quenching rate constant (*K*_sv_) and fluorescence quenching rate constant (*K*_q_) were calculated by the Stern–Volmer formula [21]:

(1) $$F_{0}/F=1+K_{\mathrm{SV}}\left[Q\right]=1+K_{q}\tau_{0}\left[Q\right]$$

where *F*_0_ and *F* are the steady-state fluorescence intensities in the absence and presence of quencher, respectively; *K*_sv_ is the Stern–Volmer quenching rate constant; *K*_q_ is the quenching rate constant of Hb; τ_0_ is the average lifetime of the protein without the quencher being equal to 10^−8^ s; and [*Q*] is the concentration of quencher.

For the static quenching, the binding constant (*K*_a_) and number of binding sites (*n*) may be calculated as following equation:

(2) $$\log\left[\left(F_{0}-F\right)/F\right]=\log K_{a}+n\log\left[Q\right]$$

*K*_a_ and *n* can be obtained by the intercept and slope of the double logarithm regression curve of log [(*F*_0_ − *F*)/*F*] versus log [*Q*], respectively.

As shown in Table 1, the values of *K*_q_ at three temperatures were markedly greater than the collisional quenching constant (2 × 10^10^ M^−1^ s^−1^) [22]. These results demonstrated that the fluorescence quenching of protein was induced by the binding changes rather than collisional quenching and thus indicated the formation of ground state complexes between protein and drug. The unchanged *K*_sv_ for temperature rising ascertained this result [23]. Accordingly, the value of *K*_a_ could be calculated by Equation (2). The *K*_a_ of the complexes was 1.6 × 10^6^ L/mol at 298 K. Generally, a value of *K*_a_ greater than 1 × 10^4^ L/mol indicated a robust interaction between protein and drug [21]. Even at the higher temperature of 308 K, the *K*_a_ was still greater than 1 × 10^4^. Collectively, these results demonstrated strong interaction between the protein and the drug.

### 3.2. Preparation and Characterization of ANSs

**Figure 2:**
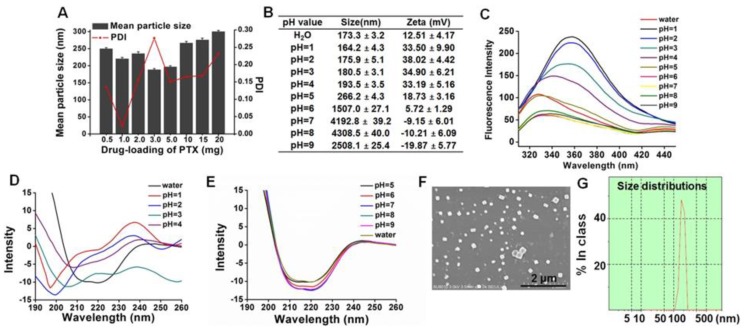
(**A**) The particle size with the drug loading of PTX from 0.5–20 mg. (**B**) Particle size and zeta potential of ANSs with the pH value ranging from 1–9. (**C**) The fluorescence emission spectrum of Hb with pH from 1–9. The far UV Circular Dichroism (CD) spectrum of Hb at pH (**D**) ranging from 1–4 or (**E**) ranging from 5–9. (**F**) The scanning electron microscopy (SEM) image and (**G**) size distribution of ANSs with 50% loading of PTX.

ANSs were prepared by an anti-solvent precipitation method followed by ultrasonic treatment. First, the effect of drug loading from 5% to 200% (compared to the weight of protein, *w*/*w*) on the particle size of ANSs was investigated (Figure 2A). All ANSs had a diameter of <300 nm; while <200 nm-ANSs were from the formulations with drug loadings of 30% and 50%.

Second, the effect of pH on the particle size of ANSs with drug loading of 50% was investigated (Figure 2B). Interestingly, under acidic conditions at pHs of 1–5, the particle size was significantly smaller than that at pHs of 6–9; additionally, the absolute values of potential from ANSs prepared under acidic conditions were generally greater than that at pHs of 6–9; Furthermore, under acidic conditions, decreasing the pH led to a reduction of the particle size of ANSs with drug loading of 50%, along with a reduction from 266 nm to 164 nm. Obviously, the acidic conditions, in particular pHs of 1–4, facilitated the formation of ANSs. It has been reported that in acidic conditions the globular protein is unable to maintain its rigid structure, becomes expandable and hollow, and, consequently, has greater capability to adsorb other molecules [24,25]. Accordingly, the increased adsorption of hydrophobic drug into the protein could induce changes in the protein’s structure and the microenvironment of intrinsic residuals. To prove our assumption, fluorescence and CD spectra were performed. As depicted in Figure 2C, significantly, the maximum fluorescence intensity at around 340 nm from pHs of 1–5 was greater than that of pHs of 6–9. These results implied that the Trp residues of protein were embedded in a stronger hydrophobic condition and, in turn, demonstrated the improved adsorption of hydrophobic drug under acidic conditions. Additional examination of CD spectroscopy demonstrated that pHs of 1–4 induced red shift from around 220 nm–200 nm and pHs of 1 and 2 enabled increased negative minimum at approximately 200 nm compared with that of water (Figure 2D); however, pHs of 6–9 had little effect on the negative minimum (Figure 2E). The results indicated that acidic conditions could change the secondary and tertiary structure of the protein and that decreasing the pH from 4 to 1 allowed for more profound alteration. Collectively, we initially identified that the potential reason the acidic condition improved the drug loading and facilitated the formation of ANSs was due to the changes of the protein’s structure.

Overall, ANSs with drug loadings of 30% and 50% had a particle size less than 200 nm; however, the latter had higher drug loading and was selected for further study. SEM examination revealed that the ANSs had a tetragonal structure with a size of 150–250 nm (Figure 2F), being in line with the DLS results (Figure 2G).

### 3.3. Amorphous State and In Vitro Drug Release

**Figure 3:**
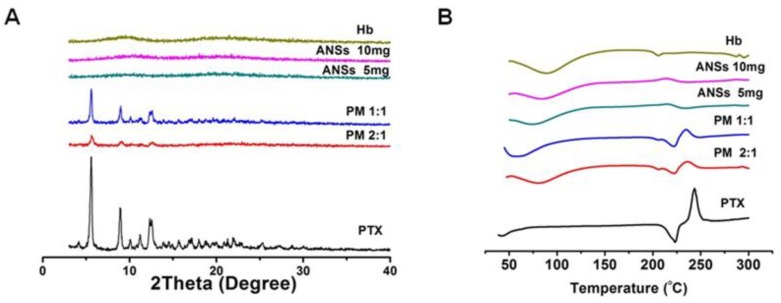
(**A**) Physical state of the drug in ANSs analyzed by PXRD and (**B**) differential scanning calorimetry (DSC). PM 1:1 and 2:1 indicate physical mixture (PM) of Hb/PTX at mass ratios of 1:1 and 2:1.

To study the drug state in ANSs, examinations of PXRD and DSC were performed. Pure PTX displayed characteristic crystal peaks at a 2θ angle of 5.0, 9.0, 10.0 and 12.0 [26], demonstrating high crystallinity (Figure 3A). Similar crystalline peaks were displayed in the physical mixture (PM). By contrast, no diffraction peaks of the drug in ANSs with 30% or 50% drug loading was observed, indicating an amorphous state. DSC examination presented endothermic peak of the drug at 200 °C in the samples of pure drug and PM, while this peak disappeared in ANSs (Figure 3B). Again, the DSC results indicated the drug dispersed as amorphous state in ANSs.

**Figure 4:**
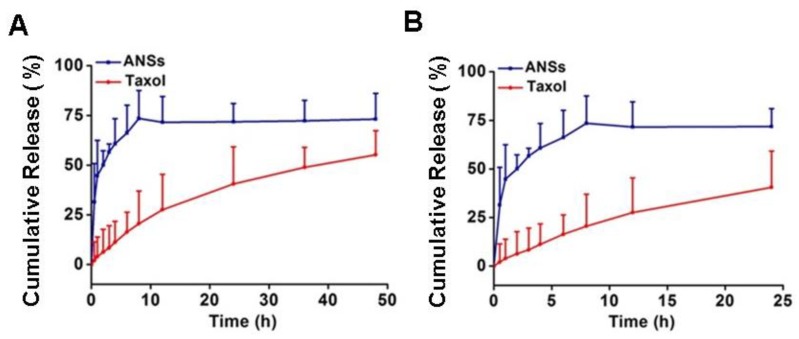
In vitro release profile of PTX from Taxol and ANSs in (**A**) a 48 h duration, and (**B**) in the first 24 h period (*n* = 5).

To further ascertain the amorphous state, test of in vitro drug release was performed. As shown in Figure 4, the ANSs exhibited significantly faster drug release even compared with the free drug formulation, Taxol. The amorphous drug possessed a higher energy state and had a higher saturation solubility and more rapid dissolution velocity [27]. Additionally, an increase in surface area due to the nanoscaled size of ANSs contributed to the faster release as well. Overall, these results confirmed the amorphous drug in ANSs.

### 3.4. Cellular Uptake and Cytotoxicity

**Figure 5:**
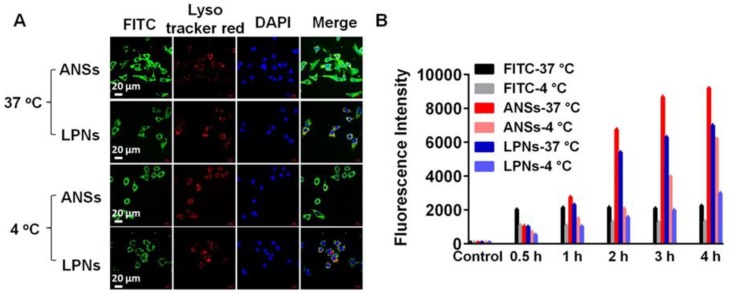
(**A**) Comparison of cellular uptake of FITC-labelled complexes of ANSs (FITC–ANSs) and FITC-labelled LPNs (FITC–LPNs) at 37 °C and 4 °C in A549 cells after 4 h incubation at a FITC concentration of 2.5 μg/mL. (**B**) Quantification of cellular uptake assayed by flow cytometry (*n* = 5).

The uptake of ANSs of PTX in A549 cells was determined with PTX nanocrystals (LPNs) as a control. Significantly, the cells exhibited stronger green fluorescence from FITC–ANSs compared to that from FITC–LPNs after 4 h incubation at 37 °C or 4 °C (Figure 5A). Quantified assay by FCM demonstrated that the uptake of these two nanoparticles was time-related in a 4 h period (Figure 5B). Importantly, the uptake of FITC–ANSs was higher than that of FITC–LPNs after 2 h incubation. The increased uptake was ascribed to the positively charged ANSs (Figure 2B) that improved the interplay between the nanoparticles and cell membrane.

**Figure 6:**
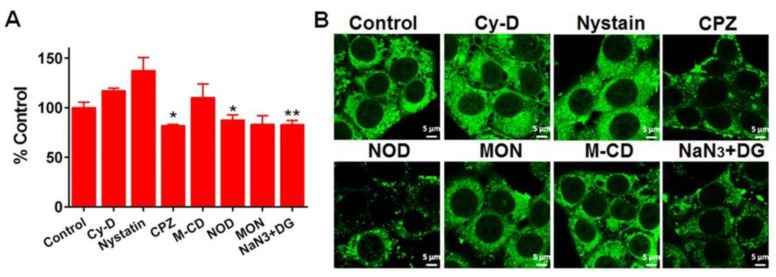
Endocytosis mechanism of FITC–ANSs in A549 cells. (**A**) Quantification uptake pretreated with cellular uptake inhibitors by flow cytometry (*n* = 5, * *p* < 0.05 and ** *p* < 0.01); (**B**) confocal laser scanning microscope (CLSM) image. The examination was performed after 4 h incubation at an FITC concentration of 2.5 μg/mL.

To determine the internalization pathway, A549 cells were pretreated with various endocytic inhibitors and then incubated with FITC–ANSs for 4 h at 37 °C. Inhibitors included Cy-D, nystatin, CPZ, NOD, M-CD, MON, and NaN_3_ + DG were involved in the study, which inhibits macropinocytosis, caveolae-mediated internalization, clathrin-mediated uptake, microtubule-related internalization, lysosome-related endocytosis, cholesterol-dependent process and energy-dependent mechanisms, respectively [28,29]. As shown in Figure 6A, the uptake of ANSs was reduced by approximately 30% by four inhibitors—CPZ, NOD, MON and NaN_3_ + DG—compared with the control group pretreated with saline. CLSM observation confirmed the results (Figure 6B). These results implied that the uptake of the nanoparticles was controlled by multi-pathway, including clathrin-mediated endocytosis, microtubule-related internalization and cholesterol-dependent process with energy-dependence.

**Figure 7:**
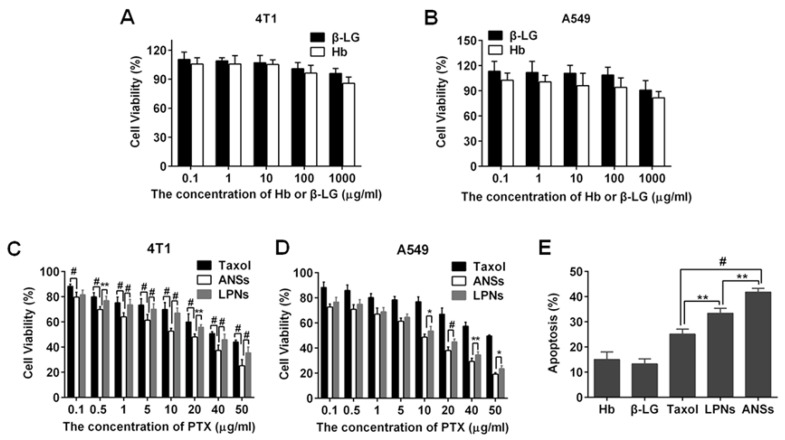
Cytotoxicity of Hb and β-LG from 0.1 to 1000 μg/mL in (**A**) 4T1 and (**B**) A549 cells. Comparison of the cytotoxicity between ANSs and LPNs with the dose of PTX from 0.1 to 50 μg/mL in (**C**) 4T1 and (**D**) A549 cells. The incubation time was 48 h. (**E**) Apoptosis rate determined by flow cytometry after 48 h incubation at a PTX concentration of 10 μg/mL at 37 °C (*n* = 3, ** *p* < 0.01 and ^#^ *p* < 0.001).

The cytotoxicity against different nanoparticles was evaluated by an MTT assay. The proteins, Hb and β-LG, had negative effect on the cell viability at a protein concentration of less than 1000 μg/mL (Figure 7A,B), demonstrating their excellent biocompatibility. The viability of ANSs after 48 h incubation was less than that of LPNs at PTX concentrations ≥0.5 μg/mL and 10 μg/mL for 4T1 and A549 cells, respectively (Figure 7C,D). An apoptotic test displayed that ANSs were able to induce the apoptosis of 4T1 cells with higher efficiency compared to LPNs (Figure 7E), increasing the apoptosis rate by 10%. Additionally, compared with Taxol, both of the two nanoparticles killed the cancer cells more significantly, along with approximately 10% increase in apoptosis rate, owing to their enhanced intracellular delivery of the drug. These results indicate that ANSs possess improved cytotoxicity over LPNs.

Releasing a considerable amount of the drug in-cell in a short period would help saturate the efflux pump and, consequently, decline the efflux of the drug and compromise the drug resistance [30]. The drug state in ANSs and LPNs was different, with the former having an amorphous state and the latter possessing a crystalline form. As displayed in Figure 4, ANSs improved the drug release significantly even compared with the Taxol; therefore, we speculated that ANSs had the potential to release their drug into cells rapidly. In contrast, it takes several hours to dissolve the intracellular nanocrystals [10,11]. Thus, we ascertained that completed drug release from ANSs would occur within a short amount of time. Indeed, ANSs caused a 10% increase in apoptosis rate compared to LPNs. Therefore, the drug state in nanosuspensions influences its pharmacological properties and, importantly, having amorphous form could enhance the activity of the drug.

## 4. Conclusions

In this study, using Hb as a carrier, <300 nm ANSs of PTX aggregated from drug-protein complexes were developed. The affinity study demonstrated robust interaction between the drug and protein. The ANSs were uptaken by cells well, and exhibited improved cytotoxicity compared with the crystalline nanosuspensions. Overall, a novel amorphous nanosuspension formulation of PTX aggregated from drug–Hb complex was developed and had the robust ability to kill cancer cells.

## Figures and Tables

**Table 1:** The binding constant of hemoglobin (Hb) and paclitaxel (PTX) in amorphous nanosuspensions (ANSs) analyzed by modified Stern–Volmer equation.

T(K)	K_sv_/(M^−1^)	K_q_/(M^−1^·s^−1^)	*n*	K_a_/(M^−1^)
288	3.5 × 10^9^	3.5 × 10^17^	0.8	7.2 × 10^7^
298	2.7 × 10^9^	2.7 × 10^17^	0.7	1.6 × 10^6^
308	1.5 × 10^9^	1.5 × 10^17^	0.5	1.7 × 10^4^
